# A severe oxaliplatin immune-induced syndrome after oxaliplatin-based pressurized intraperitoneal aerosol chemotherapy (PIPAC)

**DOI:** 10.1515/pp-2021-0138

**Published:** 2022-01-31

**Authors:** Anne-Cecile Ezanno, Brice Malgras, Olivier Aoun, Amaury Delarge, Alice Doreille, Marc Pocard

**Affiliations:** Department of Gastrointestinal and Endocrine Surgery, Bégin Military Hospital, St Mandé, France; 46th Medical Unit, 5th Armed Forces Medical Center, Strasbourg, France; Department of Acute Care Unit, Bégin Military Hospital, St Mandé, France; Department of Nephrology, Tenon Hospital, Paris, France; Department of Gastrointestinal and Cancerology, Pitié Salpetrière Hospital, Paris, France

**Keywords:** colorectal cancer, drug reaction, hypersensitivity reaction, oxaliplatin, oxaliplatin immune-induced syndrome (OIIS), PIPAC

## Abstract

**Objectives:**

Oxaliplatin immune-induced syndrome (OIIS) was recently recognized as an uncommon complication of oxaliplatin therapy.

**Methods:**

We report an exceptionally OIIS after pressurized intraperitoneal aerosol chemotherapy (PIPAC).

**Results:**

Our patient developed a severe OIIS probably related to the intraperitoneal administration of oxaliplatin. Specific tests were performed and detected high-titer antibodies to oxaliplatin.

**Conclusions:**

The OIIS is a rare. Physicians had to be aware of that clinical situation because it could be reversible, even in case of peritoneal advanced disease, and ICU treatment is justified.

## Objectives

Oxaliplatin immune-induced syndrome (OIIS) was recently recognized as an uncommon complication of oxaliplatin therapy [1]. OIIS were described in the literature after intravenous (IV) injection of oxaliplatin [2, 3]. We report an exceptionally severe multisystem organ failure shortly after a cycle of oxaliplatin-based pressurized intraperitoneal aerosol chemotherapy (PIPAC).

## Case presentation

A 39-year-old female without comorbidities was diagnosed with stage IIIc (pT4N2aM0, MSS, KRAS, NRAS, BRAF wild-type) left-sided colon adenocarcinoma in May 2016. Left hemicolectomy was performed, followed by 12 cycles of adjuvant FOLFOX chemotherapy (Oxaliplatin 85 mg/m^2^, Leucovorin, and 5-Fluorouracil) without complications. In June 2017, she had a recurrence with detection of iliac lymph nodes. She then underwent FOLFIRI chemotherapy (Irinotecan, Leucovorin and 5-Fluoruracil) combined with Cetuximab. In January 2018, an abdominal CT-scan revealed an ovarian metastasis. She underwent total hysterectomy with bilateral ovariectomy, followed by a 6 month FOLFOX chemotherapy associated with Bevacizumab. In May 2019, abdominal CT-scan revealed a local pelvic recurrence which was treated with FOLFOX chemotherapy–Panitumab protocol. In August 2020, peritoneal disease was diagnosed. She agreed to enter the PIPOX trial (phase 2). The PIPOX trial was a phase 1–2 study designed to assess the tolerance and pharmacokinetic effects of oxaliplatin administered by pressurized intraperitoneal aerosol for peritoneal metastases of gastrointestinal cancers [4]. PIPAC is a novel surgical technique for administering aerosolized chemotherapy intraperitoneally for peritoneal metastasis of various cancers. The procedure was performed under general anesthesia and capnoperitoneum (12 mmHg) using two balloon trocars placed in the midline, in accordance with the open laparoscopic technique. The aerosol was kept in a steady-state for 30 min. In October 2020, our patient was treated with a regimen of 90 mg/m^2^ oxaliplatin (160 mg) in 150 mL solution of 5% dextrose following intravenous Leucovorin 20 mg/m^2^ (72 mg) and 5-FU 400 mg/m^2^ (700 mg). No side-effects were observed. After this first operation the patient received two courses of chemotherapy as requested in the PIPOX trialSix weeks later, she returned for her second PIPAC procedure which was performed in the same manner as the first. On admission, her blood work was normal (Table 1) apart from a 24,900/mm^3^ white blood cell count secondary to the Granulocyte colony-stimulating factor administered 10 days prior per protocol. GCSF given prophylactically after chemotherapy. The next day (D1), though asymptomatic, her blood tests revealed elevated liver enzymes and acute kidney failure. She was immediately transferred to the intensive care unit for hemodialysis and close monitoring. Abdominal CT-scan did not reveal any aspect related to surgical complication. On day 2 (D2) patient underwent severe anemia and thrombocytopenia (Table 1). Direct anti-globulin test (DAT) was positive in favor of immune-mediated hemolysis. She thus received a 40 mg/day dexamethasone course during 5 days. On D5, she was transfused two units of packed red cells. Specific tests performed by the Etablissement Français du Sang (Equivalent of America’s Blood Centers) detected high-titer antibodies to oxaliplatin. By D8, due to improvement, she was transferred to a nephrology unit and switched to oral prednisone with gradual weaning. Her kidney function slowly improved and she was discharged home on D18. One month after discharge, her kidney function had returned to normal. A CT-scan showed a metastasis to Virchow’s node. Trifluridine and tipiracil (Lonsurf) combination was successfully administered.

**Table 1:** Laboratory data before, during and after hospitalization.

	Prior to admission	D1^a^	D2	D3	D8^b^	Day 19	1 month^c^
WBC/mm^3^	24,900	98,270	26,950	22,730	11,810		3,400
Platelets/mm^3^	220,000	10,700	22,000	37,000	129,000	300,000	120,000
Hb, g/dL	12.8	11.3	6.4	3.9	7	9.5	9.5
AST, IU/L	23	2,194	1,862	499	23		31
ALT, IU/L	21	1,371	1,122	723	97		33
LDH, U/L	560	>3,000			578	426	466
GGT, U/L	65	272	93	52	113		64
Total bilirubin, μmol/L	5	52	22	13	11		
Factor V		12	15	92	154		
Urea, mmol/L	3.17	17	18.7	22.1	11.6		6.51
Creatinine, μmol/L	53	354	419	591	390	426	64.6
E GFR, mL/min	115	11	10	7	12		104

^a^After PIPAC. ^b^Discharge from ICU. ^c^After discharge.

## Discussion

Oxaliplatin is the standard adjuvant treatment of high-risk stage II and III colorectal cancer [5]. A common feature of peritoneal metastasis is a reduced response to systemic chemotherapy [6]. Intraperitoneal chemotherapy is an alternative approach in treating peritoneal metastasis for improving tissue concentrations and reducing systemic toxicity. PIPAC was introduced as a new treatment for peritoneal metastases in November, 2011. Reports of its feasibility, tolerance, and efficacy encouraged centers worldwide to adopt PIPAC as a safe and promising treatment for peritoneal carcinomatosis [6]. For this purpose, the PIPOX trial, conducted by a team in Nantes, France, proposed studying oxaliplatin dose escalation during PIPAC [4]. This clinical trial determined that the maximum tolerated dose during PIPAC was 92 mg/m^2^. This study was in phase II for the evaluation of oncological outcomes. Oxaliplatin can lead to type I (with allergic hypersensitivity and IgE mediated reactions) and type II (antibody-mediated mechanism with oxaliplatin-dependent IgG antibodies) allergic and immune-allergic phenomena, respectively [7], thus being defined as the “Oxaliplatin-induced immune syndrome” (OIIS). It is well-described in the literature with an annual incidence of approximately 1/100,000 [8]. The syndrome comprises acute thrombocytopenia and/or hemolytic anemia with or without organ dysfunction, including acute renal failure and transaminitis [1]. OIIS usually developed within 24 h of oxaliplatin-based IV chemotherapy, after four or more cycles (median 16) [1]. In our case, intraperitoneal administration had a similar time-to-onset (less than 24 h after her second PIPAC) and OIIS occurred on the fourteenth oxaliplatin cycle (12 IV and 2 intraperitoneal). The initial symptoms of OIIS included fever, chills, chest or back pain, nausea, vomiting, and bleeding. As bleeding was a common but inconsistent symptom, routine blood work (as in our asymptomatic patient) to screen lactic acidosis, renal or liver dysfunctions, and altered coagulation is recommended [9]. Acute kidney injury (AKI) was less common than thrombocytopenia and anemia. It seemed result from thrombotic microangiopathy or acute hemolysis-related tubular necrosis (through hemoglobin precipitation in the renal tubules) [10] and often responded to early steroid initiation. Hemodialysis was rarely required [11]. Acute liver failure was less frequently observed [12]. It was the triggering sign in our case prompting the transfer to ICU.

The thrombocytopenia and hemolytic anemia association form the Evans syndrome (positive DAT and positive direct Coombs test on platelets) [13]. In our report, thrombocytopenia, hemolysis, and lymphopenia (352/mm^3^) occurred without neutropenia, probably because G-CSF was administered a few days earlier. The cytopenia was due to oxaliplatin-dependent antibodies to the three lineages and completely resolved with steroids. However, steroid regimen duration was not well established. The detection of drug antibodies is the best method to investigate drug-induced immune hemolytic anemia caused by the platinum family [14] because oxaliplatin antibodies can be detected in plasma of healthy blood donors by using drug-treated red blood cells. OIIS management is based on the suspension of oxaliplatin administration as soon as possible, steroids, hydration, with clinical and laboratory monitoring until recovery. The patient should not be treated further with oxaliplatin [1]. Our patient developed a severe OIIS probably related to the intraperitoneal administration of oxaliplatin. To our knowledge, this was the first case of OIIS after PIPAC with similar complications to the IV route [7]. Oxaliplatin dosage currently used during PIPAC procedures is 92 mg/m^2^ compared to the PIPOX trial phase II 90 mg/m^2^, which was very close to the IV dosage (85–100 mg/m^2^). The only difference explaining the severity of our case was the administration mode. Paradoxically, the intraperitoneal oxaliplatin administration was supposed to limit its systemic toxicity due to the barrier role of the peritoneum [4].

The OIIS is a rare, life-threatening complication of oxaliplatin therapy which might likely be more severe when administered intraperitoneally as pressurized aerosol. Physicians had to be aware of that clinical situation because it could be reversible, even in case of peritoneal advanced disease, and ICU treatment is justified.

## References

[1] Bencardino K, Mauri G, Amatu A, Tosi F, Bonazzina E, Palmeri L (2016). Oxaliplatin immune-induced syndrome occurs with cumulative administration and rechallenge: single institution series and systematic review study. Clin Colorectal Cancer.

[2] George JN, Nester CM (2014). Syndromes of thrombotic microangiopathy. N Engl J Med.

[3] Al-Nouri ZL, Reese JA, Terrell DR, Vesely SK, George JN (2015). Drug-induced thrombotic microangiopathy: a systematic review of published reports. Blood.

[4] Dumont F, Senellart H, Pein F, Campion L, Glehen O, Goere D (2018). Phase I/II study of oxaliplatin dose escalation via a laparoscopic approach using pressurized aerosol intraperitoneal chemotherapy (PIPOX trial) for nonresectable peritoneal metastases of digestive cancers (stomach, small bowel and colorectal): rationale and design. Pleura Peritoneum.

[5] de Gramont A, Figer A, Seymour M, Homerin M, Hmissi A, Cassidy J (2000). Leucovorin and fluorouracil with or without oxaliplatin as first-line treatment in advanced colorectal cancer. J Clin Oncol Off J Am Soc Clin Oncol.

[6] Alyami M, Hübner M, Grass F, Bakrin N, Villeneuve L, Laplace N (2019). Pressurised intraperitoneal aerosol chemotherapy: rationale, evidence, and potential indications. Lancet Oncol.

[7] Stack A, Khanal R, Denlinger CS (2020). Oxaliplatin-induced immune thrombocytopenia: a case report and literature review. Clin Colorectal Cancer.

[8] van den Bemt PMLA, Meyboom RHB, Egberts ACG (2004). Drug-induced immune thrombocytopenia. Drug Saf.

[9] Curtis SA, Curtis BR, Lee AI, Hendrickson JE, Lacy J, Podoltsev NA (2018). A patient with oxaliplatin immune-induced syndrome (OIIS) who also developed leucovorin and palonosetron-associated thrombocytopenia. Hematol Amst Neth.

[10] Ito I, Ito Y, Mizuno M, Suzuki Y, Yasuda K, Ozaki T (2012). A rare case of acute kidney injury associated with autoimmune hemolytic anemia and thrombocytopenia after long-term usage of oxaliplatin. Clin Exp Nephrol.

[11] Yamada S, Yazawa M, Yamamoto M, Koitabashi K, Ichikawa D, Koike J (2019). A case of biopsy-proven oxaliplatin-induced acute tubulointerstitial nephritis with thrombocytopenia and anemia. CEN Case Rep.

[12] Vyskocil J, Tucek S, Kiss I, Fedorova L, Nevrlka J, Zdrazilova-Dubska L (2019). Type II hypersensitivity reactions after oxaliplatin rechallenge can be life threatening. Int Immunopharmacol.

[13] Norton A, Roberts I (2006). Management of Evans syndrome. Br J Haematol.

[14] Leger RM, Garratty G (2011). Antibodies to oxaliplatin, a chemotherapeutic, are found in plasma of healthy blood donors. Transfusion (Paris).

